# Drowning due to vehicle falling in natural waters

**Published:** 2024-10

**Authors:** Ali Davoudi-Kiakalayeh

**Affiliations:** ^*a*^ Guilan Road Trauma Research Center, Trauma Institute, Guilan University of Medical Sciences, Rasht, Iran.

## Abstract

The Seventh National Congress on the Prevention of Drowning Cases and Vehicle Crashes in Natural Waters was held this year by the Trauma Institute of Guilan University of Medical Sciences in Iran, collaboration with universities of medical sciences nationwide. In recent years, there has been a resurgence in traffic accidents, resulting in over twenty thousand deaths this year alone. However, the number of drowning events in Iran has decreased due to various factors, despite an increase in the overall number of incidents. In Iran, approximately ten percent of drowning cases are caused by vehicles crashing into natural waters, primarily on rural roads. The main contributing factors include the lack of lighting along rural roads near waterways, roads adjacent to natural lakes and dams near residential areas, high speeds on unfamiliar driving routes, absence of roadside barriers, sudden weather changes, and heavy rainfall. Although the best way to prevent such incidents is to avoid them altogether, it is crucial to take immediate action in the first few minutes. Moreover, installing guardrails along rural roads near natural bodies of water and adding lighting to these roads are important preventive measures. Training on risk barriers on rural roads when obtaining a driving license is also key in preventing car crashes in natural waters. In the event of an accident, the driver and occupants must first remain calm and focus on an exit plan, and avoid wasting time by contacting relief organizations. The car engine should be quickly turned off to prevent future damage. Seat belts should be immediately released. If the car’s electric window system is still active, the windows should be opened quickly. If not, the car glass may need to be broken from the inside. It is recommended to break side glass first, preferably on the driver’s side. Breaking the windshield should be avoided as it can lead to water entering the car with pressure, intensifying the risk of drowning. After breaking the glass, children should be removed first, followed by older occupants. It is important to note that just two minutes after the car crashes into natural waters, there is a possibility of the car sinking completely. This is a critical time for executing an escape plan. Therefore, the driver should not waste time contacting relief organizations.

